# Neural adaptation to silence in the human auditory cortex: a magnetoencephalographic study

**DOI:** 10.1002/brb3.290

**Published:** 2014-09-30

**Authors:** Hidehiko Okamoto, Ryusuke Kakigi

**Affiliations:** 1 Department of Integrative Physiology, National Institute for Physiological SciencesOkazaki, Japan; 2 Department of Physiological Sciences, The Graduate University for Advanced StudiesHayama, Japan

**Keywords:** Habituation, magnetoencephalography, refractoriness

## Abstract

**Introduction:**

Previous studies demonstrated that a decrement in the N1m response, a major deflection in the auditory evoked response, with sound repetition was mainly caused by bottom-up driven neural refractory periods following brain activation due to sound stimulations. However, it currently remains unknown whether this decrement occurs with a repetition of silences, which do not induce refractoriness.

**Methods:**

In the present study, we investigated decrements in N1m responses elicited by five repetitive silences in a continuous pure tone and by five repetitive pure tones in silence using magnetoencephalography.

**Results:**

Repetitive sound stimulation differentially affected the N1m decrement in a sound type-dependent manner; while the N1m amplitude decreased from the 1st to the 2nd pure tone and remained constant from the 2nd to the 5th pure tone in silence, a gradual decrement was observed in the N1m amplitude from the 1st to the 5th silence embedded in a continuous pure tone.

**Conclusions:**

Our results suggest that neural refractoriness may mainly cause decrements in N1m responses elicited by trains of pure tones in silence, while habituation, which is a form of the implicit learning process, may play an important role in the N1m source strength decrements elicited by successive silences in a continuous pure tone.

## Introduction

The N1, and its magnetic counterpart N1m, response is a prominent and stable auditory evoked component with a latency of approximately 100 ms, and can be elicited by the onset (see reviews (Näätänen and Picton 1987)) and offset of sound (Hillyard and Picton 1978; Pantev et al. 1996; Yamashiro et al. 2009). Longer inter-stimulus intervals have been shown to elicit larger N1(m) responses than shorter inter-stimulus intervals (Hari et al. 1982; Imada et al. 1997; Okamoto et al. 2004; Rosburg et al. 2010). When repetitive sounds are presented, the first sound elicits the maximal N1(m) response, with subsequent sounds eliciting markedly smaller N1(m) responses (Ritter et al. 1968; Fruhstorfer et al. 1970; Fruhstorfer 1971; Budd et al. 1998).

The phenomenon of a declining N1(m) amplitude has mainly been discussed in terms of habituation and refractoriness. Habituation is derived from a psychological point of view and describes a decrement in the response as a stimulus that loses its novelty during repetitive presentations. Therefore, habituation is established progressively with sound repetition and causes gradual decrements in N1(m) responses (Thompson and Spencer 1966). On the other hand, refractoriness is derived from a neurophysiological phenomenon and describes the response decrement attributed to the refractory periods following action potentials in auditory neurons (Ritter et al. 1968). Thus, refractoriness is established immediately after the first stimulus in a repetitive sound stimuli, resulting in a larger N1(m) response by the first of the repetitive sound stimuli and similar N1(m) responses by the rest.

Budd et al. (1998) investigated whether habituation or refractoriness played a more important role in the N1 decrement using five successive 1000 Hz pure tones, and showed that the N1 amplitude decreased from the 1st to the 2nd sound stimulus, with no further decrement being observed from the 2nd to the 5th sound stimulus. Therefore, they concluded that the decrement in the N1 amplitude following stimulus repetition mainly reflected a refractory process rather than habituation. However, other studies reported clear within-train response decrements that fulfilled one of the necessary requirements for identifying this phenomenon as habituation in other studies (Thompson and Spencer 1966; Fruhstorfer et al. 1970; Picton et al. 1976).

**Figure 1:**
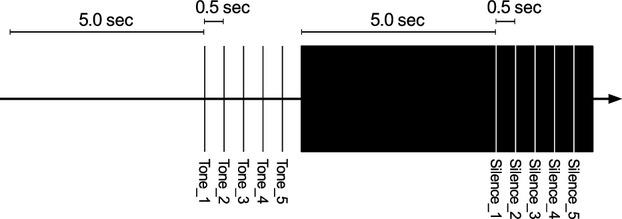
Schematic display of the auditory stimulation in the Repetitive Tone sequence (left) and subsequent Repetitive Silence sequence (right). The 1000 Hz pure tones are represented by black areas. Five successive pure tones (Tone_1, Tone_2, Tone_3, Tone_4, and Tone_5) and silences (Silence_1, Silence_2, Silence_3, Silence_4, and Silence_5) were presented with a sound onset asynchrony of 0.5 sec in the Repetitive Tone and Repetitive Silence sequences respectively. The Repetitive Tone and Repetitive Silence sequences were alternately presented and sound onset asynchrony between the two successive sequences was 7.5 sec.

Although a number of studies have demonstrated a decrement in the N1(m) response following stimulus repetition, whether decrements in the N1(m) amplitude reflect habituation or refractoriness remains controversial. In the present study, we investigated decrements in N1m responses elicited by five repetitive pure tones in silence (Repetitive Tone sequence) and five repetitive silences in a continuous pure tone (Repetitive Silence sequence) (Fig.1). A short silence with a duration of 20 ms embedded within a continuous sound has been shown to elicit an N1(m) response, the amplitude of which is influenced by the duration and intensity of the continuous sounds (Michalewski et al. 2005; Pratt et al. 2005, 2007; Campbell and Macdonald 2011). However, it currently remains unknown whether the repetitive short silences within a continuous sound decrease N1m responses. We hypothesized that neural refractoriness may be caused by a pure tone stimulation and recover during the silent intervals; therefore, neural refractoriness should be minimal for the 1st pure tone following the longest silent interval (5.0 sec) and should be maximal for the 1st silence following the longest (5.0 sec) pure tone stimulation. On the other hand, habituation should gradually decrease the N1m amplitude under both Repetitive Tone and Repetitive Silence sequences. The results of the present study may provide us with an insight into the neural mechanisms that cause decrements in the neural activity elicited by repetitive sound and silence stimuli.

## Materials and Methods

### Participants

Fifteen healthy participants (10 females; mean ± standard deviation: 23.7 ± 5.6 years) participated in the present study. All participants had normal hearing and no neurological or otological disorders. All participants were fully informed about the study and gave written informed consent for their participation in accordance with procedures approved by the Ethics Commission of the National Institute for Physiological Sciences. Therefore, the study conformed to The Code of Ethics of the World Medical Association (Declaration of Helsinki).

### Stimuli and experimental design

The experimental design is schematically represented in Figure1 [listen to Audio S1]. The test stimulus was either a 1000 Hz pure tone in silence or silence in a continuous 1000 Hz pure tone. In the Repetitive Tone sequence, trains of five successive 1000 Hz tones (Tone_1, Tone_2, Tone_3, Tone_4, and Tone_5) were presented with a sound onset asynchrony (onset-to-onset interval) of 0.5 sec. In the Repetitive Silence sequence, a continuous 1000 Hz pure tone that contained five successive silences (Silence_1, Silence_2, Silence_3, Silence_4, and Silence_5) with a sound onset asynchrony of 0.5 sec was presented. The 1000 Hz pure tone in the Repetitive Tone sequence had a duration of 25 ms, including 5-ms onset and offset ramps. Each silence in the Repetitive Silence sequence also had a duration of 25 ms, including 5-ms offset and onset ramps. Each sequence had a duration of 7.5 sec, the Repetitive Tone and Repetitive Silence sequences were alternately presented, and the first sequence was randomly chosen. Tone_1 and Silence_1 appeared 5 sec after the offsets of the Repetitive Silence and Repetitive Tone sequences respectively. All sounds were diotically presented through plastic tubes 1.5 m in length and earpieces fitted to the participant's ears. Before starting magnetoencephalography (MEG) data acquisition, each participant's hearing threshold for the 1000 Hz pure tone was individually determined for each ear. During the MEG recording session, the repetitive pure tones and continuous pure tone in the Repetitive Silence sequence were presented at an intensity of 45 dB above individual sensation levels. In order to keep the participants alert and distracted from the auditory signals, a self-chosen silent movie was presented during the MEG recordings. Each MEG measurement consisted of 200 Repetitive Tone sequences and 200 Repetitive Silence sequences, resulting in 200 epochs per condition.

### Data acquisition and analysis

Auditory evoked fields were measured with a helmet-shaped 204-channel whole head planar-type gradiometer (Vector-view, ELEKTA, Neuromag, Helsinki, Finland) located in a silent, magnetically shielded room. The signals were passed through a 0.3–200 Hz band-pass filter and digitized at 600 Hz. The evoked magnetic fields were averaged selectively for each condition, starting 100 ms prior to the onset, and ending 250 ms after the offset. Epochs containing amplitude changes greater than 3 pT were discarded as artifact-contaminated epochs.

The source locations and orientations of the auditory evoked fields were estimated using BESA software (BESA Research 5.3.7, BESA GmbH, Germany). To analyze the N1m component, the averaged auditory evoked fields were 30 Hz low-pass filtered (zero-phase shift Butterworth filter, 24 dB/oct), and the baseline was corrected relative to the 100 ms pre-stimulus interval. No significant differences were noted in the estimated N1m locations between the conditions, which was consistent with previous studies reporting no significant source location difference between the N1m-on and N1m-off responses (Pantev et al. 1996; Yamashiro et al. 2009). Therefore, we averaged the magnetic fields of all conditions to improve the signal-to-noise ratio and used these averaged magnetic waveforms to estimate the single equivalent current dipoles reflecting the N1m response. Initially, the peak N1m response was identified as the maximal root-mean square value of the global field power approximately 100 ms after the test stimulus onset. Source locations and orientations were then estimated based on the 10-ms time window around the N1m peak by means of a single equivalent current dipole model for each participant and each hemisphere individually.

The dipole locations and orientations were determined in head coordinate systems with the origin set to the intersection of the medial–lateral axis (*x*-axis) between the pre-auricular points of the left and right ears with the posterior–anterior axis (*y*-axis) running through the nasion, and the inferior–superior axis (*z*-axis) through the origin perpendicular to the (*x*–*y*-plane).The estimated sources, which had a fixed location and orientation for each hemisphere in each participant, served as a spatial filter (Tesche et al. 1995) during the calculation of the source strength waveforms to obtain the maximal N1m source strength and latency in each hemisphere and each condition.

To evaluate the N1m source strength decrements elicited by the repetitive tones and silences, the source strength of the N1m elicited by the 2nd, 3rd, 4th, and 5th test stimulus was normalized with respect to the N1m source strength elicited by the 1st test stimulus for each participant and each hemisphere in each sequence (Repetitive Tone or Repetitive Silence) individually. Normalization was used in order to reduce the impact of inter-individual and inter-sequence variabilities in the N1m source strength. The normalization procedure was not applied to the N1m latency due to the comparably small variability of latency among participants and sequences. Normalized N1m source strengths were evaluated by means of a repeated-measures analysis of variance (anova) using three factors: SEQUENCE (Repetitive Tone vs. Repetitive Silence), HEMISPHERE (Left vs. Right), and POSITION (2nd, 3rd, 4th, and 5th) in the sequences. In order to investigate the effects of POSITION on each sequence (Repetitive Tone or Repetitive Silence), the normalized N1m source strengths were separately evaluated in each sequence by means of a repeated measures anova using HEMISPHERE (Left vs. Right) and POSITION (2nd, 3rd, 4th, and 5th) as factors. The normalized N1m source strength elicited by the 1st test stimulus was left out of the statistical analysis because we mainly focused on the N1m decrement patterns from the 2nd to 5th test stimulus in order to investigate refractoriness and habituation effects.

N1m latencies were also evaluated by means of a repeated-measures anova using the three factors, SEQUENCE (Repetitive Tone vs. Repetitive Silence), HEMISPHERE (Left vs. Right), and POSITION (1st, 2nd, 3rd, 4th, and 5th). In the present study, the *P* values provided for repeated-measures anova results were Greenhouse–Geisser corrected and Bonferroni corrected pairwise *t*-tests were performed for post hoc multi-comparisons.

## Results

**Figure 2:**
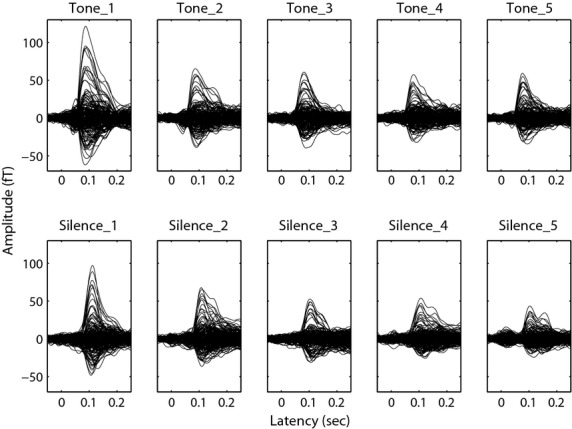
Auditory evoked magnetic fields of one representative participant elicited by five successive pure tones (upper panels: Tone_1, Tone_2, Tone_3, Tone_4, and Tone_5) and five successive silences (lower panels: Silence_1, Silence_2, Silence_3, Silence_4, and Silence_5). Clear N1m responses could be observed at a latency of approximately 0.1 sec.

**Figure 3:**
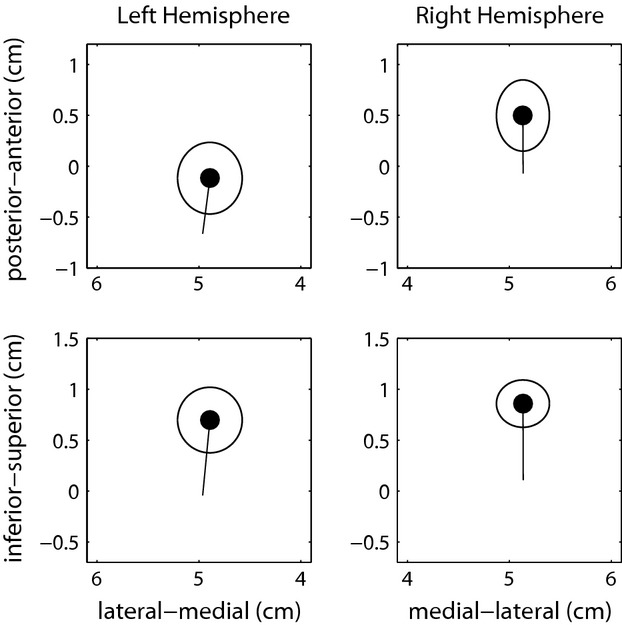
Estimated source locations (circles) and orientations (lines) of the N1m responses in the *x*-*y* plane (medial–lateral vs. posterior–anterior directions) and *x*–*z* plane (medial–lateral vs. inferior–superior directions). The ellipses around the circles denote the 95% confidence intervals.

The mean number of trials remaining after artifact rejection was 197.6 (standard deviation = 4.8) and clear auditory evoked N1m responses were obtained under each condition (cf. Fig.2). In the present study, we concentrated on the N1m component because the P1m and P2 m responses obtained were markedly smaller than the N1m response (cf. Fig.2) and the generator sites of the P2m were unclear (Godey et al. 2001). Figure3 displays the mean dipole locations and orientations of the N1m responses with the 95% confidence intervals. The goodness-of-fit of the underlying dipolar source models for the averaged MEG waveforms was above 90% in all participants (mean ± standard deviation: 95.75 ± 2.0%). The estimated dipolar sources were located at the superior temporal plane, which corresponded to the N1m generator as reported previously (Pantev et al. 1995; Eggermont and Ponton 2002).

### N1m cortical source strength and latency

**Figure 4:**
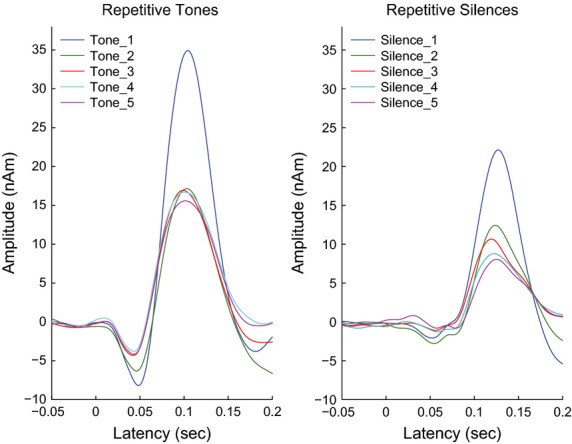
Time courses of the mean source strengths across all participants (*N* = 15) and hemispheres in the Repetitive Tone (left) and Repetitive Silence (right) sequences. Each colored line represents each condition (see legends in the left upper corner).

The time courses (time range from −50 to + 200 ms) of the source strengths grand-averaged across all participants and hemispheres are displayed in Figure4. The N1m responses had larger amplitudes and shorter latencies in the Repetitive Tone sequence than in the Repetitive Silence sequence.

**Figure 5:**
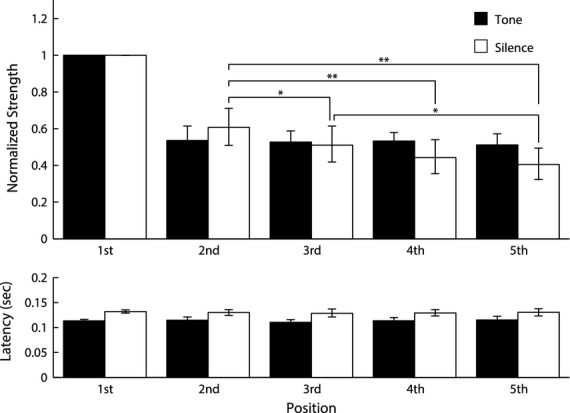
Group means (*N* = 15) of the normalized N1m source strengths (upper graph) and N1m latencies (lower graph) in each position of the five successive stimuli (1st, 2nd, 3rd, 4th, and 5th) including error bars denoting the 95% confidence intervals. Filled and open bars denote the Repetitive Tone and Repetitive Silence sequences (*P <* *0.05, ** *P *<* *0.001 [Bonferroni-corrected]).

Figure5 shows the mean normalized N1m source strengths and N1m latencies in each condition together with the corresponding 95% confidence intervals obtained by boot-strap resampling tests (iteration = 100 000). The three-way repeated-measures anova evaluating normalized N1m source strengths resulted in a significant main effect for POSITION (*F*_(3, 42) _= 5.66, *P *<* *0.02) and a significant interaction between SEQUENCE and POSITION (*F*_(3, 42) _= 7.46, *P *<* *0.001). No significant main effect was detected for SEQUENCE (*F*_(1, 14) _= 0.53, *P *=* *0.48) or HEMISPHERE (*F*_(1, 14) _= 0.07, *P *=* *0.79). The significant interaction between SEQUENCE and POSITION appeared to indicate that the effects of sound repetition on N1m decrements differed between the Repetitive Tone and Repetitive Silence sequences.

The two-way repeated-measures anova evaluating the normalized N1m source strengths in the Repetitive Silence sequence resulted in a significant main effect for POSITION (*F*_(3, 42) _= 9.40, *P *<* *0.001), but not for HEMISPHERE (*F*_(1, 14) _= 0.02, *P *=* *0.88). The post hoc multi-comparison test detected significant differences between Silence_2 and Silence_3 (*t*_(29) _= 3.01, *P *<* *0.04), Silence_2 and Silence_4 (*t*_(29) _= 4.62, *P *<* *0.001), Silence_2 and Silence_5 (*t*_(29) _= 5.24, *P *<* *0.001), and Silence _3 and Silence_5 (*t*_(29) _= 3.26, *P *<* *0.02). The two-way repeated-measures anova evaluating the normalized N1m source strengths in the Repetitive Tone sequence resulted in no significant main effect for POSITION (*F*_(3, 42)_ = 0.18, *P *=* *0.78) or HEMISPHERE (F_(1, 14)_ = 0.11, *P *=* *0.75). The normalized N1m source strengths gradually decreased from the 2nd to the 5th silence in the Repetitive Silence sequence, whereas the normalized N1m source strengths remained constant from the 2nd to the 5th in the Repetitive Tone sequence (Fig.5).

The three-way repeated-measures anova evaluating N1m latency resulted in a significant main effect for SEQUENCE only (*F*_(1, 14) _= 124.73, *P *<* *0.001). The N1m latency was more prolonged in the Repetitive Silence sequence than in the Repetitive Tone sequence; however, the N1m latency was similar between hemispheres and between different positions in both the Repetitive Tone and Repetitive Silence sequences.

## Discussion

The results of the present study demonstrated that the decrement patterns of the N1m responses elicited by repetitive sound stimulations depended on the sound types (Repetitive Tone vs. Repetitive Silence). The source strengths of the N1m responses decreased from the 1st to the 2nd in both the Repetitive Tone and Repetitive Silence sequences (Fig.5); however, we did not observe further N1m source strength decrements over positions from the 2nd to the 5th in the Repetitive Tone sequence, while a gradual but significant decrement in the N1m source strength was noted from the 2nd to the 5th in the Repetitive Silence sequence.

Exposure to repetitive sounds with identical features is known to induce a decline in auditory evoked N1 and N1m response amplitudes in a silent environment. This phenomenon has often been explained in terms of habituation and refractoriness (Budd et al. 1998). Habituation is an implicit learning neural process that causes the modulation of auditory evoked responses according to the relevancy of the corresponding sound inputs (Sokolov 1963; Thompson and Spencer 1966). On the other hand, refractoriness is a single cell-based neurophysiological mechanism that reflects the recovery cycles of the stimulated sensory neurons (Ritter et al. 1968; Rosburg et al. 2010). The N1m response amplitude is known to be influenced by both top-down and bottom-up neural inputs (Okamoto et al. 2011). Therefore, it is difficult to disentangle response decrements associated with habituation and those associated with refractoriness. One of the methods used to distinguish the neural processes related to habituation or refractoriness is to investigate the time course of response declines (Barry et al. 1992; Budd et al. 1998). Habituation is established progressively as the stimulus is repeated and, thus, loses its novelty for listeners. Therefore, the effect of habituation on the N1m response is characterized by a gradual decrease in the response according to the repetition of sound stimuli. In contrast, refractory mechanisms elicit a fast drop in the response strength from the first to the second stimulation, with no further decline being observed from the second stimulation onwards because the effect of refractoriness mainly depends on the recovery cycle, namely the silent interval between the preceding and corresponding test stimuli.

While some previous studies reported progressive N1(m) decrements elicited by repetitive sound stimuli, thereby supporting the habituation mechanism (Thompson and Spencer 1966; Fruhstorfer et al. 1970; Öhman et al. 1972), most studies observed no further N1(m) decrement from the 2nd stimulation, which is characteristic of refractoriness (Ritter et al. 1968; Budd et al. 1998; Rosburg 2004). The present results obtained in the silent condition (Repetitive Tone sequence) also support the refractoriness mechanism because they show that N1m source strengths became significantly smaller from Tone_1 to Tone_2, followed by no further decrement (Fig.5). In the Repetitive Tone sequence, the degree of recovery from refractoriness caused by the preceding tone appeared to contribute to the N1m source strengths elicited by the repetitive tones. The silent intervals preceding the tone played a major role in the N1m response amplitude.

In the present study, the results observed in the Repetitive Silence sequence showed significant gradual N1m source strength decrements from the 2nd to the 5th, implying the contribution of the habituation mechanism (Fig.5). Although decrements in auditory evoked N1m responses elicited by repetitive pure tones in silence have been examined extensively, information on repetitive silences is limited. Previous studies (Pratt et al. 2005, 2007; Palmer and Musiek 2013) demonstrated that silent gaps with a duration of 20 ms in continuous white noise could elicit clear auditory evoked responses. In the present study, we used a continuous 1000 Hz pure tone and five successive silent gaps with a duration of 25 ms (5-ms onset and offset ramps) as test stimuli and obtained clear auditory evoked N1m responses, as shown in Figure2. A previous study (Lanting et al. 2013) investigated the effects of the duration (100, 225, 475, or 975 ms) of the preceding 1000 Hz pure tone (adapter) on the N1-P2 response elicited by the 1000 Hz pure tone probe with a duration of 100 ms that was presented 25 ms after the offset of the adapter. The findings obtained revealed that the longer the duration of the adapter, the larger the reduction in the N1–P2 response elicited by the adapted sound (probe). However, in the present study, we observed the largest N1m response elicited by Silence_1, which followed the longest adapter (5.0 sec). The bottom-up driven neural refractoriness caused by the continuous pure tone stimulation did not appear to play an important role in the Repetitive Silence sequence in the present study. The main reason may be that the probe response in the previous study (Lanting et al. 2013) was derived by subtracting the adapter alone response from the response to the adapter and probe pair. This procedure may have eliminated the auditory evoked responses elicited by the adapter offset. The longer duration of the sound stimuli could elicit larger auditory N1-off responses (Hillyard and Picton 1978; Hari et al. 1987). In the present study, the offset N1m responses elicited by the 5.0 sec continuous 1000 Hz pure tone may have overlapped with the onset N1m responses elicited by the subsequent 475 ms 1000 Hz pure tone. Though the offset N1m response caused by the preceding sounds could explain the N1m source strength difference between Silence_1 and Silence_2, the gradual N1m source strength reduction from Silence_2 to Silence_5 cannot be explained by refractoriness or stimulus offset responses. Moreover, Pratt et al. (Pratt et al. 2007) investigated the effects of preceding noise durations (0.5, 1.5, 2.5, and 4.0 sec) on the N1a and N1b components elicited by 20 ms silent gaps and observed that the peak amplitudes of both N1a and N1b were not significantly affected by the preceding noise duration. Therefore, it is less likely that the N1m response elicited by silence merely represents overlaps in the N1m-off response elicited by the pure tone preceding the silence and the N1m-on response elicited by the pure tone following the silence.

As shown in the stimulus paradigm (Fig.1), we replaced the silent intervals and pure tones in the Repetitive Tone and Repetitive Silence sequences. In the Repetitive Tone sequence, silence and pure tones constituted the background and foreground respectively. The role of the background and foreground may not be determined by the sound property itself, but rather by the relevancy and relative frequency of the sound inputs. In the Repetitive Silence sequence, the continuous pure tones occupied most of the acoustic environment, while silence rarely appeared. The continuous pure tones may be irrelevant in the Repetitive Silence sequence and may also act as the background. In contrast, the silence may be segregated as a relevant figure from the background and may also play a principal role in auditory neural processing. The repetition of the silences may decrease its novelty, which could initiate a neural habituation process, leading to gradual N1m source strength decrements, as observed in the present study (Fig.5). Another possibility is that the time course of the habituation process may differ between Repetitive Tone and Repetitive Silence sequences. Knowledge and experience of sound stimulus natures have been shown to attenuate human auditory evoked cortical responses (Schafer et al. 1981; Pantev et al. 1998; Zacharias et al. 2012). We are frequently exposed to sound sequences similar to the Repetitive Tone sequence in daily life, (e.g., the tick-tock of a clock); however, repetitive short silences within a continuous steady sound similar to the Repetitive Silence sequence are hardly heard. Familiarity with a Repetitive Tone sequence may make it possible to evoke a strong and saturated habituation effect from the 2nd pure tone, whereas several trials may be needed to assess the silences as irrelevant sound signals in the unfamiliar Repetitive Silence sequence, leading to gradual N1m decrements from the 2nd to the 5th in the Repetitive Silence sequence.

The N1/N1m decrements may also be attributed to neural activity decrements in the periphery, namely a forward masking effect in the cochlea. Previous studies demonstrated that a longer preceding masking sound caused smaller neural activity by the following probe, and the effects of forward masking disappeared after 200 ms in the auditory nerve (Harris and Dallos 1979; Shore 1995). Therefore, the forward masking effect in the cochlea may have no influence on the Repetitive Tone sequence. The forward masking effect in the cochlea may decrease the N1m response in the Repetitive Silence sequence; however, in the present study, the N1m response was the largest in Silence_1, which followed the longest tone causing the largest forward masking effect. Therefore, the forward masking effect in the cochlea could not explain the results obtained.

In conclusion, the results of the present study suggest that both refractoriness and habituation mechanisms are involved in decrements in auditory evoked N1m responses. Under quiet circumstances, in which silence occupied most of the time, listeners could use the silent period to allow auditory neurons to recover from refractoriness. In such a case, refractoriness appeared to mainly cause the auditory evoked N1m decrement. In contrast, under noisy environments, in which irrelevant sound signals occupied most of the time, auditory neurons may not have a silent interval long enough to recover from refractoriness. In such a situation, less frequent auditory events (e.g., silence) appear to be processed as salient auditory signals. If they were presented repeatedly, they would gradually lose their novelty and the corresponding neural activity would be decreased. Therefore, habituation appears to play a more important role in the Repetitive Silence sequence than in the Repetitive Tone sequence.

## Supporting Information

**Audio S1.** An exemplary sound representing repetitive tone sequencing and subsequent repetitive silence sequencing as shown in Figure 1.
