# Supplementary material for: Neural adaptation to silence in the human auditory cortex: a magnetoencephalographic study
Source: Brain Behav. 2014 Sep 30;4(6):858–66. doi: 10.1002/brb3.290 (PMC4212114; doi:10.1002/brb3.290)
Supplement: Supplementary file 2 [file brb30004-0858-sd2.docx]

An exemplary sound representing repetitive tone sequencing and subsequent repetitive silence sequencing as shown in Figure 1.
